# Sticky Tunes: How Do People React to Involuntary Musical Imagery?

**DOI:** 10.1371/journal.pone.0086170

**Published:** 2014-01-31

**Authors:** Victoria J. Williamson, Lassi A. Liikkanen, Kelly Jakubowski, Lauren Stewart

**Affiliations:** 1 Hochschule Luzern – Musik, Lucerne University of Applied Sciences and Arts, Luzern, Switzerland; 2 Department of Music, University of Sheffield, Sheffield, United Kingdom; 3 Helsinki Institute for Information Technology HIIT, Aalto University, Espoo, Finland; 4 Psychology Department, Goldsmiths, University of London, London, United Kingdom; Northwestern University, United States of America

## Abstract

The vast majority of people experience involuntary musical imagery (INMI) or ‘earworms’; perceptions of spontaneous, repetitive musical sound in the absence of an external source. The majority of INMI episodes are not bothersome, while some cause disruption ranging from distraction to anxiety and distress. To date, little is known about how the majority of people react to INMI, in particular whether evaluation of the experience impacts on chosen response behaviours or if attempts at controlling INMI are successful or not. The present study classified 1046 reports of how people react to INMI episodes. Two laboratories in Finland and the UK conducted an identical qualitative analysis protocol on reports of INMI reactions and derived visual descriptive models of the outcomes using grounded theory techniques. Combined analysis carried out across the two studies confirmed that many INMI episodes were considered neutral or pleasant, with passive acceptance and enjoyment being among the most popular response behaviours. A significant number of people, however, reported on attempts to cope with unwanted INMI. The most popular and effective behaviours in response to INMI were seeking out the tune in question, and musical or verbal distraction. The outcomes of this study contribute to our understanding of the aetiology of INMI, in particular within the framework of memory theory, and present testable hypotheses for future research on successful INMI coping strategies.

## Introduction

Many everyday thoughts are spontaneous, meaning they appear to be unrelated to the task at hand and that their instigation is not under conscious voluntary control [1], [2]. It is estimated that up to 40% of everyday thoughts fall into the category of spontaneous cognitions [3]. This form of mental activity is exemplified by phenomena such as mind wandering [4], [5] and involuntary autobiographical and semantic memories or ‘mind pops’ [6], [7].

One of the most commonly reported forms of everyday spontaneous cognition is involuntary musical imagery (INMI) or, colloquially, ‘earworms’. These terms describe the spontaneous recall and replay of musical imagery within the *mind’s ear* [8] that goes on to repeat on an involuntary loop. INMI is a ubiquitous phenomenon that over 90% of people report experiencing at least once a week [9] with higher prevalence among individuals who play and sing music regularly and who see music as an important part of their daily lives [9]–[12].

There are few reliable situational predictors for the onset of an INMI episode. INMI may be more prevalent in states of extreme high or low cognitive load [13] however, the majority of studies have reported more coincidental triggers for INMI, such as recent exposure to music or memory associations with chance meetings, sights or sounds [10], [14], [15]. This evidence implies that INMI could occur in a broad range of situations, a factor which may go some way to explaining the phenomenon’s prevalence [16].

Given the omnipresence and frequency of INMI in everyday life [9], [17], it is reasonable to ask how people react to the experience. For the purposes of the present paper we consider that ‘INMI reactions’ comprise two components: how people feel about the experience (‘INMI evaluations’) and the actions they perform in response to the experience (‘INMI behaviours’).

The literature on INMI reactions to date has focused largely on INMI evaluations. The majority of INMI episodes are rated as neutral or pleasant, whereas around a third of people rate their experience as disturbing or annoying [9], [10], [14], [18]. Disturbing INMI can lead to distraction, anxiety or upset [19]–[21]. These negative reactions are similar to those noted for other forms of spontaneous cognition such as mind wandering, which can be associated with impaired attention [22] and increased subjective unhappiness [23].

By comparison, little is known about INMI behaviours, despite the fact that there have been interesting anecdotal descriptions by individuals [19]. Furthermore, what is known about INMI behaviours may be confounded by a lack of information about the relationship between INMI evaluations and subsequent behaviours. The only large scale survey of INMI reactions [10] found that the majority of individuals use musical distraction behaviours to ameliorate the experience (46.67%). However, in a subsequent diary study from the same paper the majority of INMI met with a passive behavioural response (56%), such as letting the imagery dissipate rather than actively attempting to control cessation. These contradictory findings between active and passive behavioural responses hint at a level of complexity in INMI behaviours that may partly be driven by INMI evaluations.

The efficacy of INMI behaviours is also of particular interest. Beaman and Williams [10] conducted a post hoc analysis of their diary data and reported that active distraction strategies such as trying to hum another tune were associated with longer INMI episodes compared to passive strategies such as letting the imagery dissipate on its own. This evidence suggests that active behaviours are unlikely to be successful at abating INMI. Despite this finding, there are many anecdotal descriptions that people successfully use active behaviours to manage their INMI, which implies that the present literature has yet to uncover the true diversity of INMI behaviours. Direct exploration of the efficacy of INMI behaviours may also provide unique insights into the origins of this form of everyday, spontaneous thought.

In summary, although research to date has explored INMI evaluations and, to a lesser extent, the types and efficacy of INMI behaviours, the relationship between these factors remains poorly understood. We set out to collate and categorise a large sample of reports on INMI in order to better appreciate the full range and complexity of INMI reactions, both evaluations and behaviours. The research questions that framed the present study were: I) how do people react to INMI? (Study 1 and 2), II) do INMI evaluations influence INMI behaviours? (Study 1), and III) are INMI behaviours effective? (Study 2).

In order to collate a substantial database on INMI reactions we took advantage of two existing online INMI surveys, one in Finnish (N = 12519) [9], [24] and one in English (N = 5989) [15]. Although the two surveys were designed separately, with differences in overall emphasis and scope, the commonalities between them were such that related questions could be asked of each survey and overarching themes could be teased out via a combined analysis on a subset of the data from both surveys. The main difference between the two studies was while the Finnish study contained data on INMI evaluations and behaviours, the English study comprised data on INMI behaviours and their efficacy.

The qualitative method chosen for the present study of INMI reactions was inspired by grounded theory analysis [25]–[27]. Grounded theory is an appropriate approach to take when reactions in a situation are likely to vary widely and there is therefore a need to synthesise and categorise a large amount of data. The chosen method [15] prescribed that the research was not driven by specific hypotheses; rather, the objective was to uncover consistent patterns within the data and to develop emergent theories regarding how people react to INMI episodes (evaluations and behaviours) and the efficacy of any response behaviours.

Grounded theory is a progressive form of thematic analysis that begins with initial research questions. Responses from participants that relate to these questions are then categorised into themes and a descriptive presentation of the core content is formed, in this case using visual models. Finally, theories regarding the outcomes of the data are developed, upon which hypotheses for future empirical work may be derived. By this inductive process the present paper moved from initial research questions regarding INMI reactions through to a thorough categorisation of INMI evaluations and behaviours. Finally, we considered potential explanations for the resulting categorisation and proposed testable hypotheses to inform the origins of INMI and their potential control.

## Results

### Study 1: Finnish Survey

The responses to the prompt “*Have you ever done any of the following because of the music that is playing in your head*?” are detailed in Table 1. The most popular INMI behaviours were production of sound (speech or music based), followed by other distraction activities such as listening to TV or radio, and finally ‘engaging with’ the INMI tune, for instance by singing the melody.

**Table 1:** List of limited response options from the Finnish study (Study 1), translated from the original Finnish.

Have you ever done any of the following because of the music that is playing in your head?	Proportion
Hum, sing or talk aloud	74.6%
Try to figure out the identity of the song	60.2%
Listen to the particular song	57.3%
Listen to music, radio or television to prevent songs playing	50.5%
Sing or play the particular song	40.7%
Try to focus on doing something else	29.5%
Avoid listening to music	0.0%

**Figure 1:**
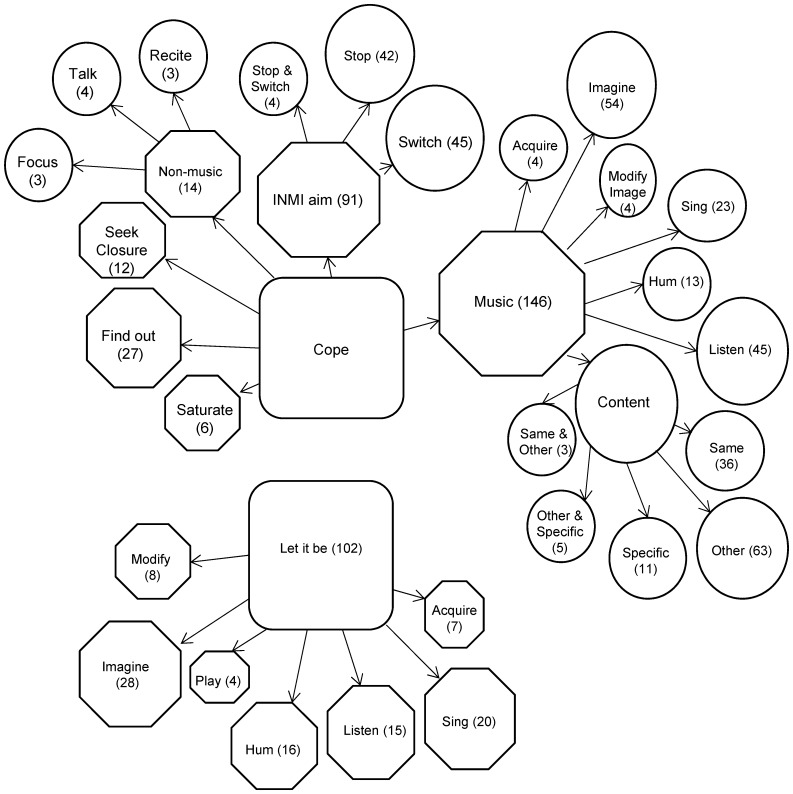
Visual model of all INMI reactions including evaluations and behaviours (Finnish Study). This figure shows all the themes generated from analysis of the Finnish INMI Survey corpus. The main dominant themes are shown in boxes, second level themes are shown in octagon shapes, and lower themes are illustrated with rounded shapes. The numbers within all themes indicate the number of codes assigned and their size is designed to illustrate relative magnitude where possible (not to scale). Hierarchical links between dominant and sub-dominant themes are illustrated with directional arrows. Themes are only shown in the model if there were more than three occasions where they were coded.

The qualitative analysis of the open text responses from the question “*What more would you like to tell us about earworms*?”can be seen in Figure 1. The responses are presented in the form of a visual model that represents the main themes and any associated hierarchical relationships. Within the data there were a small number of comments regarding the avoidance of music that may trigger INMI (N = 10) but these were not included in the main model as they reflected proactive INMI behaviours, which were not an interest of the present paper.

Figure 1 illustrates that there were two main categories of INMI behaviours, one based on negative INMI evaluations and one based on a positive appraisal of the experience. The main INMI behaviour theme that emerged from negative INMI evaluations was termed ‘Cope’, and referred to activities aimed at removing the INMI including many forms of music distraction (e.g. ‘Imagine’, ‘Sing’ and ‘Listen’), non-musical activities (‘Talk’), and activities related to engaging with the INMI tune (‘Saturate’ and ‘Seek closure’). The INMI behaviour theme related to positive INMI evaluation was termed ‘Let it be’; within this theme there were several behaviours that emerged from enjoying interaction with INMI, including playing, humming or singing along to the music. Thus the open text responses from Study 1 confirmed that choices about INMI behaviours were often pre-empted by an individual’s evaluation of their INMI experience.

#### ‘Cure’ tunes

Twelve people (5.59% of participants) reported that they used a specific song as a form of music distraction for their INMI, a behaviour we termed ‘Cure’. Nine cure tunes were identified and two were unspecified. Only one song, Kashmir by Led Zeppelin, was mentioned twice. The rest of the songs were mixed Finnish classics, including children’s rhymes, and English vocal rock and pop music.

### Study 2: English Survey

Responses to the question “*When I want to get rid of my earworms I normally…”* are detailed in Table 2. The most popular INMI behaviour was ‘Let my mind wander’, followed by general or musical forms of distraction. The most popular option overall, selected by nearly half the sample (43.68%), was ‘Other’, a finding which indicates that the limited response options failed to capture a large proportion of INMI behaviours.

**Table 2:** Limited option responses from the English Study (Study 2).

When I want to get rid of my earworms I normally…	N	Proportion
Try to suppress them	651	10.87%
Try to distract myself with another song	692	11.55%
Try to distract myself by thinking about something that is likely to hold my attention	533	8.90%
Let my mind wander	1321	22.06%
Other	2616	43.68%
N/A – I never try to get rid of earworms	176	2.94%

**Figure 2:**
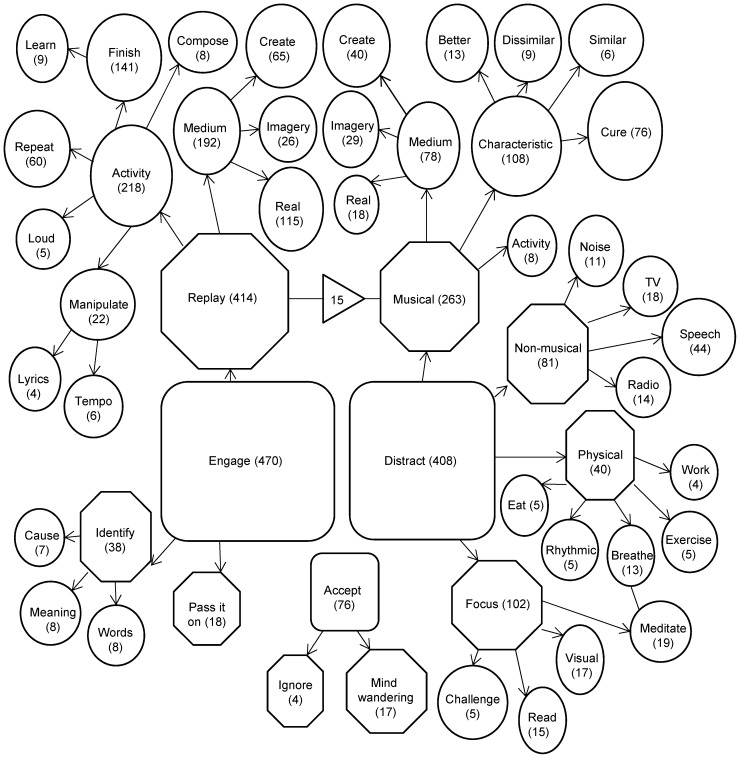
Visual model of all INMI behaviours (English Study). For details of the structure please see the legend from Figure 1. In addition: The only example of an association between themes of the same level (between ‘Replay’ and ‘Distract’- ‘Musical’) is indicated with a straight line. The number in a triangle across this associative link shows the number of occasions when two themes were coded together, and the direction of the triangle illustrates the direction of this association.

The qualitative analysis of the 831 open text responses that emerged from the open-ended follow-up question (“*If you selected ‘Other’…”*) can be seen in Figures 2 (all responses) and 3 (only those responses deemed to be effective in controlling INMI; see Table S1 for terms). In this data there were comments regarding ‘Stopping’ (N = 12) or ‘Suppressing’ earworms (N = 3; *I just stop thinking about them*). These comments were not included in the final model as they were minimal and because they did not contain enough detail to extract a type of INMI behaviour. Similarly there were a small number of comments regarding avoiding music that may trigger INMI (N = 4; *I have best success by avoiding music altogether*). These comments were also not included because they express a proactive method rather than an INMI behaviour.

Both figures illustrate that there were three main categories of INMI behaviour: ‘Accept’, ‘Engage’ with the INMI tune, and ‘Distract’ by a multitude of forms (‘Musical’, ‘Non-musical’, ‘Physical’ and ‘Focus’). Participants also provided information about reasons or purposes of replaying INMI tunes (‘Activity’) and the method by which they listened to either the INMI tune or other music (‘Medium’).

#### ‘Cure’ tunes

**Figure 3:**
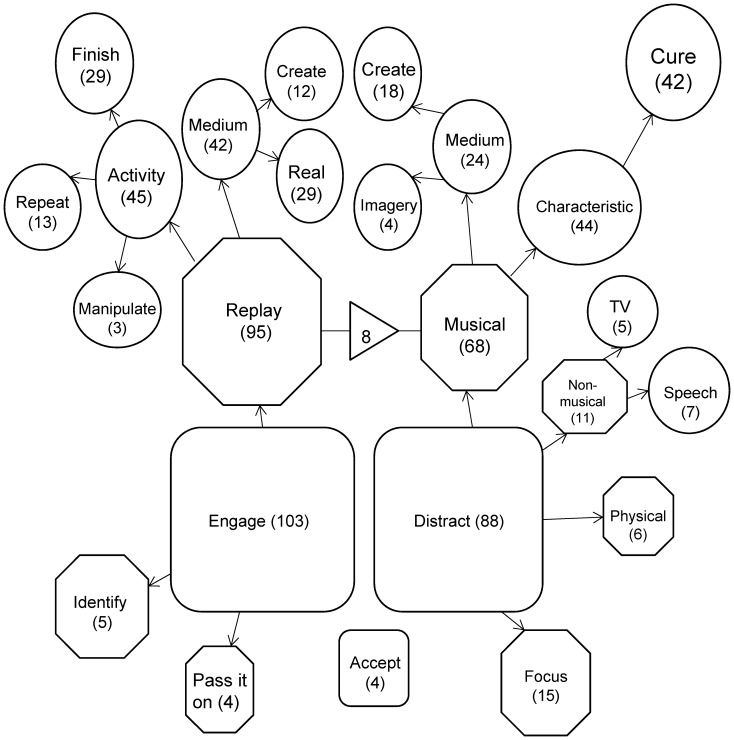
Visual model of all INMI behaviours (English Study) that were rated as ‘effective’. For details of the structure please see the legends from Figures 1 and 2.

There were 76 reports of the theme ‘Cure’ in Figure 2 (2.26% of the total codes in Figure 2), where an individual stated that they repeatedly used a certain song to control their unwanted INMI (42 in the ‘effective’ model; 5.63% of total codes in Figure 3). In total, 64 different tunes were identified, of which 6 were named by more than one participant. These six tunes are listed in Table S2. In the majority of cure tunes cases (N = 70) participants suggested that these tunes interfered with the experience of INMI but did not themselves adopt the characteristic of INMI, namely the involuntary replaying in the mind’s ear. The remaining six cases mentioned that the tune could become INMI but in these cases the individual preferred to have the cure tune in their mind compared to the original unwanted INMI.

### Combined Analysis

Combined analysis of the two main models from the Finnish survey (Figure 1) and the English survey (Figure 2) was carried out by two of the authors (VJW and LAL). The aim of this final analysis stage was to summarise the common themes between the models and thereby extract the most common INMI behaviours from the reports.

Once again, it is important to note that the open text question from the Finnish survey asked about INMI in general while the English survey promoted participants to focus on behaviours that they adopt to control unwanted INMI. This meant that some items from the Finnish survey, in particular those that related to positive INMI evaluations (‘Let it be’), had no parallel in the English survey. In the combined analysis, we therefore compared only behaviours in response to unwanted INMI. We discuss additional themes regarding INMI evaluations and behaviours in the Discussion. Overall, there were several points of congruence across the two surveys concerning the meta-themes of ***Distraction*** and ***Engagement***, which we discuss below.

#### Distraction

Both models featured INMI behaviours that were aimed at drawing attention away from the INMI and towards another stimulus (‘Distract’ or ‘Cope’) and these themes could be musical or non-musical in nature. In both models themes such as ‘Distract- Musical’ (English) or ‘Switch’ to other music (Finnish) were more popular than themes that described the replacement of INMI with a non-musical distraction. Both models also featured the use of targeted musical distraction, a ‘Cure’ tune that the participants used to combat their unwanted INMI experiences. Where non-musical themes were reported these were biased towards verbal content such as conversation, recital (including prayer and meditation), watching TV or reading.

#### Engagement

In both models participants reported that they voluntarily chose to engage with (listen to) the INMI tunes that were stuck in their minds (‘Replay’- English: ‘Same’- Finnish). Moreover, people from both samples reported the need to listen to the INMI tune more than once in an attempt to exhaust the mental representation of the music (‘Repeat’- English: ‘Saturate’- Finnish). A common reason for INMI replay behaviours was to listen to the whole tune in order to complete the small section of music that featured as INMI (‘Finish’- English: ‘Seek Closure’- Finnish). Furthermore, many people sought to learn some unknown aspect of the tune that they felt may be causing it to repeat (‘Identify’- English: ‘Find out’- Finnish).

## Discussion

Involuntary musical imagery (INMI) is part of a wider compendium of experiences known as spontaneous cognitions or ‘mind pops’ [7]. These include autobiographical memory recollections [6] and mind wandering [4], [5], [29]. The present study provides an opportunity to focus on three aspects of INMI that are poorly understood: the effects (evaluations), response to the effects (behaviours), and outcome of response (efficacy).

INMI is a regular experience for the majority of people [9], [17]. Many report that they either enjoy or feel ambivalent towards these episodes [9], [10], [14], [18] although these same people can often also recall INMI experiences where their reaction was annoyance, distraction or even distress [19], [21]. The present study corroborates these generic observations, as both types of reaction were found in the reported data.

The aim of the present research was to analyse a substantial body of reports on INMI reactions, in order to further elucidate INMI evaluations (positive and negative) and response behaviours, as well as the relations between them. We conducted two independent qualitative analyses of INMI reactions gathered from two online surveys. The analyses were driven by the following questions: I) how do people react to INMI? (Study 1 and 2), II) do INMI evaluations influence INMI behaviours? (Study 1), and III) are INMI behaviours effective (Study 2)? Our thematic conclusions provide answers to these questions as well as providing a grounded theory basis for future empirical hypotheses testing of INMI origins and control. These hypotheses are discussed below along with the respective consideration of the research questions.

In terms of overall reactions to INMI, we confirmed that while some people describe positive evaluations of the experience (Study 1), a number report displeasure towards their INMI episodes (Study 1 and Study 2). This variety in reactions is to be expected given previous literature [9], [10], [14], [18], [19], [21] however, in contrast to previous work we found that subsequent active INMI behaviours can be effective in ameliorating the experience (Figure 3). This result challenges earlier conclusions about the dynamics of INMI coping. Beaman & Williams [10] (p.647) stated that ‘*according to [Wegner’s theory of ironic mental control], any conscious attempt to displace or remove an earworm is unlikely to succeed as monitoring this attempt simply re-presents the earworm to the individual’*. The results of the present study refute the suggestion that attempts at monitoring unwanted INMI necessarily re-trigger the experience and prevent INMI control.

Given that people report both positive and negative evaluations of INMI and that they feel able to control their INMI if necessary, we now go on to consider first, the role of evaluations in INMI reaction and second, the efficacy of particular INMI behaviours.

Firstly, the question of whether INMI evaluations impact on INMI behaviours is addressed by comparing passive (as opposed to active) INMI behaviours across our models. In the Finnish study the theme ‘Let it be’ described situations where participants reported enjoying their INMI, an evaluation more likely to be met with a passive behavioural response. We propose that the occurence of passive INMI behaviours is therefore dependent on whether or not an individual wishes to purge the experience. Considered in this way, enjoyment (‘Let it be’) and/or passive acceptance were among the most popular overall INMI reactions across both models in the present paper, in line with the survey carried out by Beaman & Williams [10]. This finding supports the idea that the majority of INMI experiences are not bothersome [9], [14], [18].

A second point of interest with regard to the link between INMI evaluations and behaviours is the finding that the behaviours like ‘Listen’, ‘Sing’ and ‘Imagine’ all occurred in response to both positively and negatively evaluated INMI (Study1). This result indicates the importance of noting the relationship between INMI evaluation and behaviour, as a similar behaviour can follow a quite different evaluation of the experience. We can only speculate as to why a similar behaviour is adopted in both positively and negatively evaluated INMI. The data suggests that while ***Engagment*** with the INMI tune was thought by some people to be effective for combating unwanted INMI (possible reasons are discussed below), it was also a response that was primed when people enjoyed the music in their heads.

With the importance of considering the relationship between INMI evaluation and behaviour noted, we now go on to consider the behaviours that people consistently adopted when INMI was evaluated as bothersome and unwanted, and in particular, the efficacy of these coping behaviours. The meta-themes from the combined analysis are considered in the order in which they were presented: ***Distraction*** and then ***Engagement***.

The open text reports (Figures 1, 2 and 3) highlighted the extent of the INMI ***Distraction*** behaviours that people adopt, which included musical, verbal and visual tasks, physical activity (exercise and breathing techniques), challenging mental pursuits, or re-focusing attention. The sheer breadth of these INMI behaviours extends well beyond those recorded by previous limited option response studies, a result which advocates for diverse and open questioning of INMI reactions as opposed to restricted sampling.

The features of effective ***Distraction*** provide clues as to the origins of unwanted INMI. The most effective forms were musical, followed by verbal (conversations, TV, recital). Visual or spatial activities featured sparsely by comparison and not at all as effective behaviours. These dynamics of INMI ***Distraction*** can be interpreted in terms of memory theory. Unitary memory frameworks such as the Object-Oriented Episodic Record (O-OER) model [30], [31] and Feature Model [32] predict that competition in short-term memory is highest when stimuli streams have similar characteristics. Within the framework of the working memory model [33], memory for music is processed in a similar way to verbal materials, as distinct from visual or spatial memory [34]–[36]. These theoretical frameworks are all congruent with the pattern of effective responses seen here, where music, followed by sequential sounds (speech), are reported to be more distracting to INMI compared to visual or spatial tasks. Interestingly, the finding that people experience less INMI when completing a verbal as opposed to a visual task [13] would also be predicted by these models.

In light of these theories and initial findings, future investigations of the role of memory in INMI origins should compare systematic, controlled levels of musical, verbal and visual ***Distraction*** on INMI experiences, balancing for executive load and individual differences, in order to further elucidate which memory systems are involved in the generation of INMI. Such investigations could also provide valuable insights into the successful control of unwanted INMI once it has begun.

Finally, concerning INMI ***Distraction***, both of the present studies noted the use of ‘Cure’ tunes whereby participants employed a particular song to control INMI. In most reports this tune blocked the involuntary cycle of INMI without itself adopting this repeating characteristic. This is the first known report of such a phenomenon within INMI studies. Some ‘Cure’ tunes received multiple reports, even across the two datasets, suggesting they may possess general validity as an aid to INMI cessation. However, the majority were named by only one person. It will be for future empirical analysis to determine whether these tunes possess musical or verbal characteristics that lend them to function as INMI cures more generally and support efforts at INMI control.

The second identified meta-theme of effective behaviour for dealing with unwanted INMI was ***Engagement***, whereby people listened to or played the tune that was stuck in their heads. This behaviour was reported as a way to complete an unknown or un-experienced aspect of the INMI tune. This finding concurs with reports that the majority of INMI features short segments or snippets of larger tunes [10], [13], [14] and suggests, in addition, that people seek to extend this fragment of INMI in order to resolve unwanted INMI experiences.

The reports of ***Engagement*** are in line with the emergent theory that some episodes of INMI occur as a result of a Zeigarnik effect. Zeigarnik theory states that intrusive thoughts are triggered by the sensation that those same thoughts are incomplete and it is this incompleteness that results in them being retained for longer in memory [37]. In the case of INMI, most individuals report knowing only a portion of the original music very well [13], thereby fulfilling one of the main components for an incomplete and intrusive stimulus thought pattern; this pattern was observed in the present data.

One study to date found that incomplete exposure to tunes was not a significant trigger for INMI as compared to hearing complete songs [13]. However, this failure to show the Zeigarnik effect as an antecedent for INMI origins does not preclude the idea that it may be a cause of the involuntary repeating, cyclic nature of the experience, which constitutes a major source for negative reactions to the phenomenon [21]. Future experimental studies of INMI control could test this Zeigarnik hypothesis by trialling a coping strategy for negatively evaluated INMI, whereby people listen to and/or learn the whole tune as opposed to only those parts of the tune that are stuck in their head.

Finally, the English survey noted a link between the themes of ‘Engage’ and ‘Distract’ relating to music, whereby people adopted a dual INMI behaviour: first listening to the INMI tune and then immediately listening to other music. This dual strategy represents a culmination of the two main active INMI behaviours. A future hypothesis could be that combining ***Engagement*** and ***Distraction*** within a short time frame may be a more effective INMI coping strategy than employing either coping strategy in isolation.

Overall, the grounded theory analysis presented in the current study builds on the tradition of thematic analysis that is increasingly popular as a method of assessing subjective responses to life events [28]. This analysis allowed us to reduce a substantial database on INMI coping to the key themes, and to extract testable hypotheses regarding INMI origins and control which were grounded in the data. Our new analysis incorporates a double coder and double coding stage format, which substantially reduces the risk of individual coder bias inherent in the standard single researcher protocol while maintaining the importance of emersion and systematic inductive open coding of qualitative data.

## Methods

### Study 1: Finnish Survey

#### Participants and procedure

Study 1 re-analysed data from a Finnish survey that was online for four months in 2007. Eleven thousand, eight hundred and thirty three participants (M age = 27.9 years, SD = 8.6, range = 8–76; 31% male) [9], [24] responded first to a limited option response regarding INMI behaviours. Participants were given an introduction to the concept of INMI and then provided with a choice of seven Yes/No responses preceded with the prompt: “*Have you ever done any of the following because of the music that is playing in your head*?” The list of response options can be seen in Table 1; participants could select ‘Yes’ to as many items as they felt appropriate. These data were tabulated and analysed using descriptive statistics.

A second source of data from the same online survey came from a single open-ended question “*What more would you like to tell us about earworms*?” This generic probe generated a large amount of information, only some of which was relevant to the present study. A post hoc screening was performed on 1229 responses which resulted in the extraction of 215 comments (17.49%) that referred specifically to INMI reactions. All of these open text responses were in Finnish and the participants were on average 27 years old (SD = 8.7, range = 9–73) and predominantly female (27% male). This data was submitted for qualitative analysis, in the form of thematic coding (described below), with the aim of deriving a visual model of INMI reactions.

#### Ethics statement

The study protocol was approved by the ethics committee of the University of Helsinki, Department of Psychology, Finland. Written informed consent was obtained from all participants.

#### Thematic qualitative coding

The four-stage qualitative coding method applied to the open text responses in the present study was adopted from Williamson et al. [15], a protocol that was in turn derived from descriptions of grounded theory by Payne [26] and Charmaz [25]. The coding protocol requires participation of two ‘coders’, who initially work separately and then come together to develop the final visual model. The use of two coders is a new development in grounded theory methods, which typically only require the analysis of one researcher [28]. By contrast our method of using two coders aims to minimise personal bias in the data interpretation, and to allow an initial ‘blind’ stage of coding where each individual is unaware of the likely final structure of the data.

The four-stage inductive protocol aimed to generate themes that describe INMI reactions and then display these themes in a visual model that details their relationships. ‘Inductive’ is a term used for the general approach of grounded theory and other qualitative methods (discourse and narrative analysis) that aim to derive meaning in complex data by the development of summary themes from the raw data. ‘Themes’ are single words or short phrases that capture the meaning of a longer description of an INMI reaction. Each participant report could contribute to one or more themes.

In the first stage of analysis, each of the two coders worked independently with the raw data, the text provided by participants, and used line-by-line open coding to summarise the themes within every INMI report. ‘Open coding’ is a process by which initial themes, their properties and dimensions, are identified from within the data [28]. As the coding protocol continued, each coder developed and used consistent themes where possible, only developing new ones where they were deemed necessary from new data. The coders kept detailed notes of the emergent analysis, including where and why new themes developed, and ideas about how they may eventually relate and/or combine in a larger model. This process continued until the data had been exhausted.

In the second stage of the protocol, each coder reviewed their notes and generated a complete list of the emergent themes to ‘*sort, synthesize and organize [the] large amounts of data’* that resulted from the line-by-line open coding [25] (p. 92). This stage of the analysis allowed the coders to finalise their individual themes as specifically as possible in preparation for a comparison with those generated by the other coder.

In the third stage the coders began to work together. They compared each of their themes and the definition for each theme that was detailed in their notes. As part of the protocol, each coder took a turn in explaining and justifying a theme to the other and noting how it compared to those on the other coder’s list of themes. As part of this process, final theme labels were agreed upon. This stage resembles axial coding [27] as the coders aimed to determine the degree of convergence across their two analyses and draw out one set of themes that represented both of their ideas. The vast majority of theme labels were highly similar across the two coders and required only agreement on the final theme term to be used in the next stage of analysis.

Once the final theme labels had been agreed upon, the coders compared notes on conceptions of hierarchy and patterns between the themes, and drew up a first draft visual model to indicate potential relationships between the themes.

In the fourth and final stage, the coders revisited the entire original dataset with the new, combined list of themes and recoded every report using the agreed upon final theme labels. A protocol was in place whereby in the case of disagreement during final coding, a third independent observer would judge the disputed theme assignment. However, in this analysis there were no unresolved disagreements between the coders.

Once the fourth stage of analysis was complete, the two coders drew up a visual model based on the initial model draft, which now included figures obtained from the final coding analysis (number of reports under each theme) and any additional concepts about relationships and hierarchies that had emerged from the final joint recoding. Any theme that contained less than 3 reports was not included in the final model.

### Study 2: English Survey

#### Participants and procedure

The second study comprised data that was gathered from an English language survey (*earwormery.com*) [15] that was online between 2010 and 2012. To reiterate, the data gathered from Study 2 differed from that analysed in Study 1. While Study 1 focused on INMI evaluations and behaviours in general, the present study focused on INMI behaviour and efficacy for controlling unwanted INMI.

Five thousand, nine hundred and eighty nine participants responded to the limited option response prompt “*When I want to get rid of my earworms I normally…”* Of the 5931 people who provided gender data, 2535 were male (42.74%). The mean age of the whole online sample was 35.46 years (SD = 13.45; range = 10–92). The limited option responses that were offered were based on those used in previous studies [10], and included the opportunity to chose ‘Other’. As in Survey 1 these responses were tabulated and analysed using descriptive statistics. The raw data can be seen in Table 2.

A follow-up question was then presented: “*If you selected 'Other' it suggests you have your own strategies for trying to remove your earworms. Please use the box below to let us know what they are and if they work for you or not*”. In total, 789 individuals input data into this open text field and 42 sent direct emails to the research group. Of the 789 people who provided data as part of the online questionnaire, 347 were male (43.98%). The mean age of this online sample was 36.17 years (SD = 12.86; range = 13–76).

#### Ethics statement

The study was conducted in accordance with the Declaration of Helsinki. The study protocol was approved by the ethics committee of Goldsmiths, University of London, UK. Written informed consent was obtained from all participants.

#### Thematic qualitative coding

The analysis of the open text responses to the follow-up question “*If you selected 'Other' it suggests you have your own strategies for trying to remove your earworms. Please use the box below to let us know what they are and if they work for you or not*” followed the same thematic analysis protocol as Study 1. To reiterate briefly, two researchers independently and blindly analysed the data set using a four-stage inductive protocol in order to apply themes by a process of iterative, line-by-line coding. A detailed verification of all entries was included, whereby the coders convened to inspect and agree upon the appropriate theme labels before a second complete round of coding of the data with the agreed themes began. As in Study 1 a protocol was in place to involve a third coder in cases of unresolved coding disputes, but once again this proved unnecessary as all themes were agreed.

The final output of the present study comprised two visual models, one that detailed all INMI behaviours aimed at controlling INMI, whether successful or not, and a second that detailed only those behaviours that were described as ‘effective’ by the participant. Each participant report could contain more than one theme; in total, the present analyses comprised 3357 codes, of which 746 (22.22%) were related to effectiveness. We made no assumptions about effectiveness of INMI behaviours based on the nature of the report but only coded for effectiveness when a participant offered relevant information. A table of terms from the reports was generated as the data were coded in order to ensure that decisions about the subjective efficacy of behaviours were coded consistently (Table S1). Although reports of unsuccessful and sometimes successful strategies were also coded it was not possible to create models due to insufficient data.

## Conclusions

In answer to our original questions we have found that: I) INMI reactions are diverse and complex, a pattern underestimated by previous studies, II) INMI evaluations influence choices of INMI behaviours therefore, these factors have been confounded in the past; and III) some people report being able to control their INMI.

The new methods, findings, and hypotheses in the present paper open up directions for future studies into how people may control their INMI, a phenomenon that some believe has a negative effect on their everyday lives. In focusing on the relationships between INMI evaluation, behaviour and efficacy, future studies may also generate testable hypotheses that speak to the origins and potential control of involuntary cognition phenomena in general. Most importantly, the present study has shown that people feel that they can successfully manage their unwanted INMI, if and when they determine that coping is necessary.

## Supporting Information

Table S1 **Terms used to classify INMI coping strategies by ‘efficacy’ in the English Study (Study 2).**
(DOCX)

Table S2 **INMI ‘Cure’ tunes from the English Study (Study 2).**
(DOCX)

Appendix S1 **Finnish survey themes.**
(DOCX)

Appendix S2 **English survey themes.**
(DOCX)
