# Supplementary material for: Sticky Tunes: How Do People React to Involuntary Musical Imagery?
Source: PLoS One. 2014 Jan 31;9(1):e86170. doi: 10.1371/journal.pone.0086170 (PMC3908735; doi:10.1371/journal.pone.0086170)
Supplement: Table S1 — Terms used to classify INMI coping strategies by ‘efficacy’ in the English Study (Study 2). (DOCX) [file pone.0086170.s001.docx]

**Supporting Table 1:** Terms used to classify INMI coping strategies by ‘efficacy’ in the English Study (Study 2).

| **Successful** | **Unsuccessful** | **Successful on occasion** |
| --- | --- | --- |
| All of the time  Most of the time  Majority of the time  Normally  Generally  Usually  Often  Totally  Tends to | Rarely  Never  Hardly ever  Minority of the time | Sometimes  50/50  Doesn’t always  Seems to  Occasionally |
