# Supplementary material for: Sticky Tunes: How Do People React to Involuntary Musical Imagery?
Source: PLoS One. 2014 Jan 31;9(1):e86170. doi: 10.1371/journal.pone.0086170 (PMC3908735; doi:10.1371/journal.pone.0086170)
Supplement: Table S2 — INMI ‘Cure’ tunes from the English Study (Study 2). (DOCX) [file pone.0086170.s002.docx]

**Supporting Table 2:** INMI ‘Cure’ tunes from the English Study (Study 2)

| **Name of ‘Cure’ tune** | **Artist** | **Number of reports** |
| --- | --- | --- |
| God Save the Queen  Karma Chameleon  Happy Birthday  Theme to *The A-Team*  Kashmir  Sledgehammer | Thomas Arne  Culture Club  Mildred and Patty Hill  Mike Post and Pete Carpenter  Led Zeppelin  Peter Gabriel | 6  3  3  2  2  2 |
