# Supplementary material for: Sticky Tunes: How Do People React to Involuntary Musical Imagery?
Source: PLoS One. 2014 Jan 31;9(1):e86170. doi: 10.1371/journal.pone.0086170 (PMC3908735; doi:10.1371/journal.pone.0086170)
Supplement: Appendix S1 — Finnish survey themes. (DOCX) [file pone.0086170.s003.docx]

**Supporting Appendix 1: Finnish survey themes**

The codes used in the analysis for Study 1 are presented in this appendix. Hierarchy within the model is re-represented here in the following order: upper case bold, followed by bold, then bold indented and finally, normal text. All codes are presented with the original definitions that were used during coding and an example of a representative quote from the database. The original quotes in Finnish language are presented alongside an English translation.

**LET IT BE -** Enjoying the INMI experience. Seeking musical enjoyment triggered by INMI.

**Imagine:** Enjoy the mental continuation of musical imagery –“*Good songs I like to ‘play’ in my head from the start to the end” (Hyviäkappaleita 'soitan' mielessänialustaloppuun)*

**Sing:** Enjoy singing along to the musical imagery – “*If I have a song playing in my head, I sing it aloud” (Jos mullasoijokukappalepäässä, laulansitäkohtaauseinääneen)*

**Hum:** Enjoy humming along to the musical imagery – “*Hearing music in my head leads me to humming and then many songs get mixed up” (*Musiikinpäässäsoiminenjohtaahyräilyynjasiinämeneeuseatkappaleetsekaisin)

**Listen:** Listen to the song or artist they had experienced internally *– “Songs that I like, I listen to as quickly as possible in their entirety” (Kappaleen, jostapidän, kuuntelenmahdollisimmannopeastikokonaan)*

**Modify:** Alternative ways to enjoy initially involuntary musical imagery by modifying the auditory image for instance, by changing tempo, lyrics – “*It’s funny, because I can act upon it and consciously manipulate music the way I like”* (*Hauskajuttu, koskaosaanpuuttuaasiaan, jaalkaamanipuloidatietoisestikuulemaanihaluamaanisuuntaan*)

**Acquire:** Participant reported a need to buy the music, presumably for later play back –“*seldom it’s some pleasant and interesting piece which I really like. That can lead to playing back the record or buying”(Harvemminsoijokinmiellyttäväjakiinnostavapätkä, sellainen, jostatodellapidän. Tämävoijohtaalevynsoittamiseentaijopaostamiseen)*

**Play:** Participant reported the urge to play the music on an instrument or to sing –“*Sometimes it’s pleasant and I get a very strong urge to play or sing the piece”* (*Joskuspäässäsoiminen on miellyttävääjasiihenliittyyvoimakastarvepäästäsoittamaan tai laulamaantiettyäkappaletta)*

**COPE -** People deliberately choose to remove the undesired INMI experience.

**INMI aim:** At times, coping had a clear intended outcome

**Stop:** Participant discusses ways to ‘stop’ their INMI from replaying –*“If it is a familiar song, you can ‘stop it’ by singing it from the beginning to the end” (joskappale on tuttusenvoisaada 'loppumaan' laulamallasenmielessäalustaloppuun)*

**Switch:**  INMI experience to another song – “*I learned a few mental tricks to dispel them - usually by replacing them with some more pleasant song or poem” (sittentuliopittuamuutamamentaalinenkikkaniidenkarkoittamiseksi - yleensäkorvaamallanejollaintoisella, miellyttävämmälläkappaleella tai runolla)*

**Stop and Switch:** A combination of the above

**Saturate:** Listen to the song repeatedly until some point of exhaustion is reached – “*Only cure is to listen to the same song many times in a row, so my brain doesn’t have to try to recall it. So through saturation the music stops right there” (Ainoalääke on kuunnellasamasoinutkappalemontakertaaperäkkäin, niinettäaivojeneitarvitseyrittäämuistellasitä. Näinkyllästymisenkauttasoiminenloppuukuinseinään)*

**Find out:** Person realizes there is a missing part in their mental representation of the music, lyrics, melody, harmony, etc. By discovering this missing component, they could complete their mental image and let their mind finish up with the song – “*When some song starts playing in my head, the first thing do is to figure out what it is and then listen to it until you get bored. Otherwise it never stops and that’s very irritating because it bothers you”(Kun jokualkaasoidapäässäkannattaaensinnäkinratkaistamikäkappale se on j sittenkuunnellaniinkauanettäkyllästyy.Muuten se eilopuikinäja se on hyvinärsyttävääkoska se jäävaivaamaan)*

**Seek closure:** The respondent was not missing any part in their mental representation. Instead, they thought that the internal music has incorrectly locked itself around a small segment of the whole and they should in response need to finish the song by covertly or overtly forwarding to the end of the piece – “*Music in my head usually stops if I sing the song until the end, if I try to leave it unfinished it goes on” (Päässäsoiminenloppuuyleensäkunlaulankappaleenloppuun, josyritänjättääkappaleenkeskenpäässäsoiminenjatkuu)*

**Non-music:** Non-musical activities were used for coping.

**Talk:** Discussing with somebody, possibly about the experience – “*Sometimes the song is so irritating, that I even talk about it even with my partner” (Joskusbiisi on niinrasittava, ettäkeskuskustelensiitäjopaavovaimonkanssa)*

**Focus:** Concentrating intently on something else than music – “*Only if I’m focused on something very demanding thinking, does the music stop”*(Vain, josolenhyvinkeskittynyterittäinvaativaanajatteluun, soiminenlakkaa)

**Recite:** Recital of rehearsed or written texts, mantras or poems to occupy mind on something else – “*I’ve noticed that you can stop the music by repeating a mantra or some simple phrase” (Olen huomannutettäpäässäsoimisensaaloppumaantoistamallajotakinmantraataiyksinkertaistahokemaa*).

**Music:** Music is used for coping with INMI.

**Content:** Participant describes the musical content they use for coping. The options are *Same, Other or Specific.* Same refers to the song initially experienced as INMI. Other is any song but the Same. Specific is a dedicated piece of music the person uses in an attempt to terminate their INMI – *“If the song is irritating enough, you must use a flush song, the March of Jägers works second best, to replace the irritating song” (Jos kappale on tarpeeksiärsyttävä, pitääkäyttäähuuhtelukappaletta, jonaJääkärienmarssitoimiitoistaiseksiparhaiten, syrjäyttäenärsyttäneenkappaleen)*

**Imagine:** Mentally playback a song – *“I try to eradicate them (if they irritate) by intentionally thinking about some better song” (koitanpäästäniistä (joslaulualkaaärsyttää)*

**Listen:** Playback the recording – *“The best way to eradicate a song is to listen it from a record in its entirety” (Paraskeinopoistaabiisipäästä on kuunnella se levyltäkokonaan*)

**Sing:** – “*I then always try to drive away by singing some good song that I like” (Pyrinainasittenkarkoittamaanbiisinpoisalkamallalaulaajotainhyvääkappaletta, jostapidän)*

**Hum:** - “*By humming Fire by Jimi Hendrix you can get any song out of your mind and end up with Fire” (jimihendrixinfireähyräilemälläsaaminkäbiisintahansamielestäjatilallejää (ikäväkyllä) se Fire)*

**Modify image:** - “*I focus more on the rhythm than on words, so it gets less irritating and stops eventually” (keskitynsilloinenemmänmusiikinrytmiinkuinsanoihin, jolloinärsyttävyyslaimeneejapäässäsointiloppuujossainvaiheessa.)*

**Acquire: -***“Then you need to get it on your computer and after few days of listening, you don’t feel like ever hearing it again” (Sitten se on pakkohankkiakoneelleja kun sitä on muutamanpäivänkuunnellu, ei tee mielienääkoskaankuullakyseistäkappaletta)*
