# Supplementary material for: Sticky Tunes: How Do People React to Involuntary Musical Imagery?
Source: PLoS One. 2014 Jan 31;9(1):e86170. doi: 10.1371/journal.pone.0086170 (PMC3908735; doi:10.1371/journal.pone.0086170)
Supplement: Appendix S2 — English survey themes. (DOCX) [file pone.0086170.s004.docx]

**Supporting Appendix S2: English survey themes**

All the codes used in the final analysis for Study 2 are shown below. The logic of presentation is equal to the Finnish survey themes format (Appendix 1).

**ENGAGE** – “*Trying to engage with the song in this way works sometimes*”

**Replay song:** The individual triggers a replay of the earworm - “*Listen to the music in question*”

**Medium:** The individual identifies the source of the earworm

Create*:* Singing/humming/playing instrument - “*I find examples of that song & sing along*”

Imagery:A mental experience of the music – “*Try to play the entire song through in my head*”

Real*:* Music from personal devices. Uses of the terms ‘physical’ or ‘actual’ were accepted as references for real music- “*I listen to the actual piece of music*”

**Activity:** The individual specifies something to be done with the music – “*Go to a PC or mp3 player and listen to the song stuck in my head at least 3 times*”

Repeat*:* The earworm must be heard more than once – “*Listen to the actual song on repeat*”

Finish: The earworm must be heard in full to the end – “*I listen to the song in full*”

Learn: The earworm must be learnt or a fault corrected – “*I usually have to learn the song in its entirety before it will leave my brain*”

Manipulate: The features of the earworm are altered - “*Try to vary it - add notes, change tempo, improvise*”: Includes Tempo/lyrics: “*Slowing the music down as if you've put a 45 on 32 rpm and then speeding it back up*”/ “*I like to work with my earworms creatively--change lyrics, alter the musical bridge, etc*”

Compose: The earworm must be written down or recorded – “*Compose or recall a musical resolution or coda for the thing that's playing*”

Loud: The replay of the earworm is loud - “*Play the song on youtube in semi open headphones very loudly*”

**Identify:** The individual seeks to identify an element in the earworm – “*Identifying the earworm tends to make it go away*”

**Words:** “*Learn the lyrics of the song - usually works*”

**Cause:** “*Work out what the tune is then ponder why it might have appeared - answering those two usually gets it gone*”

**Meaning:** “*Ask if there is a message to this inner music. I usually work out what it's about”*

**Pass it on:** The individual performs the earworm with the hope that it becomes an earworm for other people – “*sing the song out loud and pass the ear worm to someone else*”

**DISTRACT** – “*Combination of trying to distract myself with another song or something likely to hold my attention*”

**Musical:** The individual uses music to distract from the earworm – “*listening to music outside my head always works*”

**Medium:** The individual identifies the music source

Create*:* Singing/humming/playing instrument - “*I'll play something else on guitar for a while*”

Imagery: A mental experience of the music – “*I sing another, specific song in my head until it goes away*”

Real: Includes personal devices and computers –“*Listen to another song (on an iPod, etc.)*”

**Characteristic:** The individual specifies a distinctive property or feature of the musical distraction – “*I sing another, specific song in my head until it goes away*”

Cure: The distraction is chosen because it will block the earworm and not become an earworm itself- “*play the lic from Layla by Eric Clapton in my head. It gets rid of whatever song I had there before but it doesn't actually stick in there*”

Better: The distraction is preferred over the present earworm –“*Sing a song I like better to override it. Works”*

Similar: The distraction must have similar features to the earworm – “*I confuse the earworm with a similar tune*”

Dissimilar: The distraction must have dissimilar features to the earworm – “*I listen to another piece of music, of a different style than that of what is looping in my mind*”

**Activity:** The individual specifies something to be done with the distraction music – “*I find it sometimes helps to play a different song that I am familiar with and stop it halfway through - my brain then seems to try to complete the new song and that helps it stick*”

**Non musical:** The individual reports using sound to block the earworm but does not overtly mention music – “*I have talk radio on - NOT MUSIC. If music comes on I have to race and turn it off*”

**Speech** (vocal and subvocal)**:** “*I try listening to spoken word instead*”

**Radio:** “*I listen to random radio for 10 mins*”

**TV:** “*distract myself with the television or cooking*”

**Environmental noise:** “*Try to relax or focus on different noises, both externally (i.e rain or clock ticking) and internally (i.e tinnitus or breathing)*”

**Physical:** The distraction activity involves body movement – “*Try to do something physical rather than mental*”

**Eating:** “*eat liquorice or something with a strong flavour it can distract the brain*”

**Exercise:** “*Go for a run so hard that I can't think about anything - usually works”*

**Work:** “*Get busy doing my work or other chore*”

**Breathe:** “*I try concentrating on my breathing and counting the breaths*”

**Rhythmic:** “*Doing something else moderately rhythmic seems to distract me enough to make it go away*”

**Focus:** Mentions either the word ‘focus’ or a task that inarguably requires focus – “*I try harder to focus on the work at hand*”

**Mediation** (chanting, prayer)**:** “*Mind clearance techniques from yoga and meditation*”

**Challenge** (maths, computer games)**:** “*I focus my attention on numbers, probability, science or reading a book about these subjects*”

**Reading:** “*If able, I often turn on the radio, the TV and open a book*”

**Visual** (imagery, drawing)**:** “*I think about drawing a picture it always works*”

**ACCEPT** – “*go with it, don't judge it, accept it*”

**Ignore:** The individual uses the term ‘ignore’; they pay no attention to the experience (as opposed to acceptance which does not presume) – “*Try to ignore it and pretend it isn't there*”

**Mind wandering:** Uses the term ‘mind wandering’; no presumption is made based on the activity – “*letting my mind wander and trying just to forget about the earworm*”
